# Maternal Experience with Predation Risk Influences Genome-Wide Embryonic Gene Expression in Threespined Sticklebacks (*Gasterosteus aculeatus*)

**DOI:** 10.1371/journal.pone.0098564

**Published:** 2014-06-02

**Authors:** Brett C. Mommer, Alison M. Bell

**Affiliations:** Department of Animal Biology, School of Integrative Biology, University of Illinois, Urbana, Illinois, United States of America; University of Missouri, United States of America

## Abstract

There is growing evidence for nongenetic effects of maternal experience on offspring. For example, previous studies have shown that female threespined stickleback fish (*Gasterosteus aculeatus*) exposed to predation risk produce offspring with altered behavior, metabolism and stress physiology. Here, we investigate the effect of maternal exposure to predation risk on the embryonic transcriptome in sticklebacks. Using RNA-sequencing we compared genome-wide transcription in three day post-fertilization embryos of predator-exposed and control mothers. There were hundreds of differentially expressed transcripts between embryos of predator-exposed mothers and embryos of control mothers including several non-coding RNAs. Gene Ontology analysis revealed biological pathways involved in metabolism, epigenetic inheritance, and neural proliferation and differentiation that differed between treatments. Interestingly, predation risk is associated with an accelerated life history in many vertebrates, and several of the genes and biological pathways that were identified in this study suggest that maternal exposure to predation risk accelerates the timing of embryonic development. Consistent with this hypothesis, embryos of predator-exposed mothers were larger than embryos of control mothers. These findings point to some of the molecular mechanisms that might underlie maternal effects.

## Introduction

Non-genetic maternal effects, or the influence of a mother's phenotype on her offsprings' phenotype, are widespread across taxa [1]–[4]. Mothers can affect offspring phenotype by the level of care they give [5], and the resources they provide [6] including steroids transferred to developing offspring [7]. Maternal effects can be adaptive, conveying information about the environment that offspring are likely to encounter [2], [8] or maladaptive [3], and have been associated with human disease [9]–[10]. However we know little about the molecular mechanisms by which maternal experience influences offspring.

Studies on diverse organisms have shown that maternal stress can have long-lasting effects on offspring traits including survival [3]–[4], [8], [11], growth [4], [12]–[15], morphology [4], [14]–[16], and behavior [8], [17]–[20]. While stress-induced maternal effects can be mediated by differential provisioning of resources by mothers [2], [6], stress-induced maternal effects are often mediated by prenatal exposure to maternally-derived stress hormones. Prenatal exposure to maternally-derived stress hormones can have organizational effects on development with lifelong consequences for offspring (reviewed in [20]; [21]–[25]).

Predators are one of the most important naturally-occurring stressors for animals in natural populations [26]–[27], influencing both evolved and plastic life history traits of prey such as growth [4], [28], age at reproduction [29]–[31] and size at reproduction [31]–[32]. A number of recent studies have shown consequences of in utero exposure to predation risk [4], [8], [16], [19]. For example, predator-exposed female sticklebacks produced larger eggs with higher concentrations of cortisol and, initially, a higher metabolism [33]. Later in life, offspring of predator-exposed stickleback mothers exhibited greater antipredator behavior [33], an altered stress response to predation risk [34], and performed relatively poorly in a learning task [35]. Maternal exposure to predation risk also influenced offspring survival in sticklebacks [36]. These results are consistent with the hypothesis that maternal exposure to predation risk has organizational effects on the development of the offspring brain and HPI (hypothalamus-pituitary-interrenal) axis that influences offspring traits well into adulthood.

Despite growing appreciation of maternal effects occur across taxa and traits, we are just beginning to understand the molecular mechanisms by which maternal experience influences offspring. Therefore, we used RNA-sequencing technology to compare the stickleback embryonic transcriptomes of predator-exposed mothers and unexposed (control) mothers. We measured embryos at three days post-fertilization (dpf) because in zebrafish it is the earliest period when maternally-derived RNAs are thought to be fully degraded [37] and when brain regions begin to develop [38].

We made the following predictions about the types of genes and pathways likely to be influenced by maternal exposure to predation risk. First, because embryos from maternally-stressed stickleback mothers had higher initial respiration of oxygen [33] we predicted that maternal stress would increase transcription of yolk-processing enzymes [39] and the mitochondrial genes involved in oxidative metabolism.

Second, stress influences brain gene expression (e.g. [40]) therefore we predicted that genes associated with neural development might be differentially expressed between embryos of predator-exposed and control mothers. We also predicted that genes involved in eye development might be affected by maternal predator exposure because an earlier study in sticklebacks found that genes associated with eye development were differentially expressed in adults following exposure to predation risk [42].

Third, previous studies have shown that exposing fish embryos to elevated cortisol alters the expression of genes encoding insulin-like growth factor, growth hormone, thyroid hormone receptors, and growth hormone receptors [43]. Since stickleback mothers produce eggs with elevated cortisol after exposure to a predator [33] we predicted that genes that are activated or repressed by cortisol or synthetic glucocorticoids would be differentially expressed between treatments. Three day post-fertilization stickleback embryos do not produce endogenous cortisol (Paitz et al, unpublished). Therefore we did not expect that the key gene products required for HPI axis function, such as corticotropin-releasing hormone (CRH), adrenocorticotropic hormone (ACTH), and proopiomelanocortin (POMC) to be expressed. Also, since glucocorticoids are known to affect the expression of immunity-related genes, for example genes of the complement pathway (reviewed in [44]), we predicted an effect of maternal exposure to predation risk on the expression of genes involved in immunity.

Fourth, predation pressure is associated with a relatively ‘fast’ life history (accelerated development, small size at maturity, early age at reproduction) in many taxa (fish, reviewed in [45], birds [46], amphibians [47]), including sticklebacks [48] therefore we predicted that genes related to accelerated growth and development would be upregulated in embryos as a result of maternal exposure to predation risk.

Finally, given the broad epigenetic changes that occur across the genome during tissue differentiation (reviewed in [49]), and previous studies showing that maternal care influences offspring DNA methylation patterns [41] we also predicted that genes involved in epigenetic modifications to the genome, such as the DNA methyltransferases, would be influenced by maternal exposure to predation risk.

## Methods

### Ethics Statement

This work was conducted in accordance with national standards on animal welfare as approved by the University of Illinois Institutional Animal Care and Use Committee (IACUC# 09204).

### Animal collection and maternal treatment

Juvenile threespine sticklebacks were caught by baited trap from the Navarro River, CA in July, 2011 and transported to the University of Illinois (Urbana, IL). Fish were housed until adulthood (approximately 1 year of age) in 37L tanks (53L x 33W x 24H cm) with gravel-bottom, artificial plant refugia, a flow-through system with UV, charcoal, particulate and biological filters that remove olfactory cues and a photoperiod that mimics seasonal changes at a density of 10 fish/tank. Fish were fed *ad libitum* once daily frozen bloodworms, brine shrimp, mysis shrimp, and cyclopeez.

In August, 2012 when sticklebacks showed reproductive morphology (red throat in males, gravidity in females) they were separated by sex. Males were housed individually in 9L tanks, females were housed in groups of four in 26.5L tanks (36L x 33W x 24H cm) with opaque external shading. Tanks of females were randomly assigned as control or predator exposed. The females in predator exposed tanks were chased daily for 30 seconds at a randomly chosen time with a rubber model of a sculpin (*Cottus* spp, SL: 7 cm) to simulate predation risk. Sculpin of this size are a known predator of stickleback eggs and juveniles in the Navarro River (personal observation). Females were chased 29.4±7.3 days (mean ± standard error); chased females were exposed to simulated predation risk prior to and during gravidity. Males and control females were not chased. Females were wild caught therefore it is possible that they experienced predation risk prior to the experiment, but they were randomly assigned to either the predator-exposed or control treatment. A promising future direction is to evaluate for how many generations in sticklebacks the effects of maternal exposure to predation risk persist. Further, it is possible that some of the genes differentially expressed in embryos as a result of maternal exposure to predation risk are not specific to predation risk, but instead are common to all stressors or to maternal effects in general. Future studies that expose mothers to multiple and various treatments could potentially distinguish those genes specific to predator-induced maternal effects.

### Rearing embryos

When females became visibly gravid they were netted from their tank and gently squeezed to obtain live eggs. Males were netted from their tank and immediately sacrificed with a fatal concentration (>0.2 mM) of MS-222 anesthetic. Testes were dissected from individual males, macerated, and the suspension was used to fertilize eggs in a glass petri dish. Egg fertilization was confirmed by observation under a light microscope. Clutches were obtained from n = 8 control and n = 8 predator-exposed females. Eggs were transferred to mesh-bottom cups in 9 L tanks with air bubblers directly beneath developing clutches. All embryo tanks were treated with 100 uL of methylene blue solution to prevent fungal infection. Unfertilized and infected eggs were removed daily. We sampled whole embryos at 72.040±0.012 (mean ± standard error) hours post-fertilization (3 dpf). By 3 dpf our embryos had pigmented eyes, melanopores, and intermittent tail spasms [50]. Morphological differentiation of the sexes in sticklebacks occurs no earlier than 11 dpf [51], so it was not possible to distinguish between male and female embryos. Embryos were collected between August and October, 2012. Embryos were placed in RNAlater RNA stabilization reagent (Life Technologies, Grand Island, NY) for one hour, and stored at -80C until total RNA extraction in February, 2013.

In our study gene expression was measured in whole embryos. As a result, many tissue-specific expression changes would not have been detected, particularly for genes that exhibit low levels of expression or are expressed in minute tissues. Measuring sex- and tissue-specific embryonic expression at multiple time-points is an obvious task for future study. Also, measuring cortisol in mothers, eggs, and offspring would be useful toward understanding possible mechanisms for the observed effects on gene expression, and is an obvious future direction. In this experiment it was our priority to obtain sufficient RNA from embryos for sequencing. Since the sacrifice of adult fish or viable eggs is necessary for the measurement of cortisol in sticklebacks it was therefore not possible to set aside a sufficient quantity of mothers, eggs, or offspring for steroid measurement.

### RNA Sequencing

To obtain sufficient quantity of RNA for quantitation, quality control, and template for cDNA library creation, we haphazardly selected and pooled ten embryos from each mother (n = 8 control and n = 8 experimental mothers, n = 160 embryos total). Pools represent independent mothers and so are treated as biological replicates. Embryo pools were homogenized and total RNA extracted from the homogenate using the AllPrep DNA/RNA Micro Kit (Qiagen, Hilden, Germany) according to the manufacturer's instructions (as in [52]). Briefly, each embryo pool was frozen on dry ice in a 0.5 mL microcentrifuge tube, disrupted with a pestle, treated with extraction buffer containing β-mercaptoethanol, and then homogenized with a battery operated pestle grinder (Fisher Scientific, Fair Lawn, NJ). Total RNA was purified and treated with on-column DNase digestion (Qiagen). RNA was quantified on a Nanodrop spectrophotometer (Thermo Scientific, Hudson, NH) and quality verified on an Agilent Bioanalyzer (Agilent Technologies, Palo Alto, CA). The quantity of total RNA obtained from embryo pools was 5.19±0.49 (mean ± standard error) micrograms.

For all samples cDNA libraries were constructed from 1 ug total RNA using the TruSeq Stranded RNAseq Sample Prep kit (Illumina Inc., San Diego, CA). These were quantitated on a Qubit fluorometer (Life Technologies, Grand Island, NY), run on an Agilent bioanalyzer chip, diluted to 10 nM, and quantitated again with qPCR. The library construction process selectively purified for mRNA using oligodT primers. Each of the 16 samples was individually barcoded. All samples were combined into a single pool which was divided in half and sequenced on two single-end lanes on an Illumina HiSeq 2000 (TruSeq SBS sequencing kit version 3) at the Roy J. Carver Biotechnology Center (Urbana, IL). RNA-sequencing of embryo samples produced over 356 million reads each 100 nt in length with average quality scores over 30; there were 22.28±0.48 million reads (mean ± standard error) per sample.

### Genome alignment and differential expression analysis

Raw reads were trimmed and trimmed reads aligned to the stickleback reference genome (BROADS1, Ensembl database version 71.1, Feb 2006) using TopHat 2 (version 2.0.8). Trimmed reads expressed at very low level were filtered out (described below). Across all samples 88.95±0.18 percent (mean ± stderr) of trimmed reads mapped to the genome, and 51.87±0.79 percent (mean ± stderr) that mapped to the genome mapped uniquely within known or predicted genes, consistent with other RNA-seq studies that used genomes with comparable assembly and annotation quality [53]. Genes were removed from the dataset if they were detected in only a single sample and at less than one count per million mapped reads (3339 genes) or if they had zero counts for all samples (1677 genes). The remaining 17440 out of the 22456 genes in the stickleback genome representing aligned reads were evaluated for differential expression between maternally-stressed and control embryos using two methods. First, we used the *exactTest* function in EdgeR (bioconductor.org, version 2.11). Second, we used Cufflinks/Cuffdiff (version 2.1.1). Cuffdiff employs an algorithm that probabilistically divides ambiguous fragments among genomic loci and then counts expression data at the transcript level, resulting in counts of Fragments Per Kilobase of exon per Million mapped reads (FPKM, [53]–[54]). EdgeR uses a different algorithm that counts reads at the gene level, applies a normalization factor that adjusts for library size, and reports the results as the logarithm of modified Counts Per Million mapped reads (logCPM, [53], [55]). The list of differentially expressed genes identified in EdgeR (n = 295 genes, FDR cutoff  = 0.10) was compared for overlap with the list generated in CuffDiff (n = 268 genes, FDR cutoff  = 0.05) and combined into a single dataset (Figure S1; n = 455 genes). We used different FDR cutoff values for EdgeR and CuffDiff analyses in order to offset the different algorithmic stringencies and obtain two lists with a comparable number of genes for each count method (Fig S1). The majority of genes falling between the 0.05 and 0.10 FDR thresholds in EdgeR are also significant within the Cuffdiff FDR≤0.05 threshold or they code for a different subunit of a gene that falls within the Cuffdiff threshold, confirming that our choice to use different FDR cutoffs did not greatly affect the composition of the gene list.

Stickleback Ensembl gene IDs were downloaded from BioMart (toplevel.fa version 70 ensemble.org) for all 455 differentially expressed genes along with 292 of the corresponding gene symbols (unique gene identifiers assigned by HGNC, http://www.genenames.org); 101 gene symbols for the remaining Ensembl IDs were obtained manually from the Ensembl database (release 71, Apr 2013) based on 1-to-1 orthology to other vertebrate species, for a total of 393 genes (∼86% of the dataset) with gene symbols. To visualize the general relationships between sample genome-wide expression levels we used the *plotPCA* command in R to visualize the clustering of samples on the first and second principle components.

The data discussed in this publication have been deposited in NCBI's Gene Expression Omnibus [56] and are accessible through GEO Series accession number GSE56160 (http://www.ncbi.nlm.nih.gov/geo/query/acc.cgi?acc=GSE56160)

### GO/pathway analysis

To identify enriched gene ontology (GO) categories the list of 393 genes with gene symbols was analyzed with the DAVID Functional Annotation Tool (v6.7; [57]–[58]) using the gene annotations and background genome of *H. sapiens*. A total of n = 317 of the genes with gene symbols (∼81%) were successfully recognized within DAVID. GO annotations representing biological processes (GOTERM_BP_FAT), cellular components (GOTERM_CC_FAT), molecular functions (GOTERM_MF_FAT), and KEGG pathways were considered significant with a FDR of 0.05. The DAVID analysis was carried out a second time using Human Ensembl IDs corresponding to 15504 of the 17440 detected stickleback genes (∼89%) to test the reliability of using genome as background. The majority of the 317 genes with gene symbols (>80%) were not represented in the output when Human Ensembl IDs converted from the list of detected stickleback genes were used as background, likely due to incomplete annotation of genes with official gene symbols within DAVID. Using the 17440 detected (i.e. expressed) genes as background instead of the whole genome reduced the total number of terms that met the FDR threshold but did not change the nature of the terms obtained (results not shown). Due to the fact that some gene symbols might not match between stickleback and humans and to test the effect of species-specific quality of annotation within DAVID we carried out additional analyses. The first additional analysis was run using mouse annotations and mouse genome background: 294 of 455 genes from the list of differentially expressed genes (64.6%) had corresponding gene symbols within DAVID. The second additional analysis was run using zebrafish annotations and zebrafish genome as background: 165 of the 455 differentially-expressed genes (36.3%) had corresponding gene symbols within DAVID. Finally to evaluate whether some of the identified GO terms might reflect spurious associations, a random list of n = 455 genes were selected from the total 17440 expressed genes in stickleback embryos and run through DAVID using human annotations and genome as background. This procedure was repeated three times. In all 71–75% of genes in the lists were mapped within DAVID, and all three trials produced no significant GO_BP, GO_CC, GO_MF, or KEGG pathways below the FDR = 0.05 cutoff.

A separate functional classification of the genes differentially expressed in stickleback embryos was performed using Ingenuity Pathways Analysis (IPA) (Ingenuity Systems, Redwood City, CA) using Human Ensembl Gene IDs converted from the 455 Stickleback Ensembl Gene IDs in BioMart. There were corresponding Human Ensembl Gene IDs for n = 302 of the 455 stickleback genes in the dataset (∼66%). However because the human genome has multiple orthologs per gene relative to the stickleback genome, the number of Human Ensembl Gene IDs (n = 596) we obtained was greater than the number of genes in the original dataset. Gene identifiers, their fold changes, and FDR values for the 596 human genes were uploaded to IPA and queried against the Ingenuity database to identify biological functions enriched in the dataset, at default stringency. A total of n = 334 of the 596 Human Ensembl Gene IDs (∼56%) were successfully recognized by IPA.

### Embryo morphological measurements

To understand whether changes in gene expression relate to morphological changes we measured total length and eye diameter on a subset of embryos. Clutches were thawed, decanted to glass petri dishes and photographed at 15× magnification under an Olympus SZX12 stereo light microscope with a DF Plapo 1x-PF objective and an Olympus DP26 camera. The anterior/posterior axis of each embryo (nose to tail, Fig S4) was digitally measured using the microscope's cellSens v1.6 Dimension software. Since a previous study in sticklebacks exposed to predators identified increased expression of genes associated with eye photoreceptor development [42] we predicted that maternal exposure to a predator might cause an increase in the size of the embryonic eye. To accurately measure embryo eye diameters photographs were printed and eyes measured with a ruler. The longest obtainable dimension from the area of eye pigmentation (Fig S4) of the largest visible eye of each embryo was recorded to the nearest 33.3 um. Since the physical reorientation of an embryo within the petri dish disturbed the orientation of several adjoining embryos it was not possible to obtain photographs that showed the eyes of all embryos. Therefore, we were unable to obtain dimensions of the eyes from some embryos (28%). We obtained data on embryo length from n = 127 embryos from n = 4 control mothers and n = 388 embryos from n = 14 predator-exposed mothers. We obtained data on eye diameter data from n = 92 embryos from 4 control mothers and n = 282 embryos from 14 predator-exposed mothers. Morphological data were normally distributed and were analyzed in SPSS (version 20.0) using a linear model with maternal treatment as a fixed effect and mother identity as a random effect. The model for eye diameter data was run with embryo length as a covariate.

## Results

### Overall expression patterns

**Figure 1:**
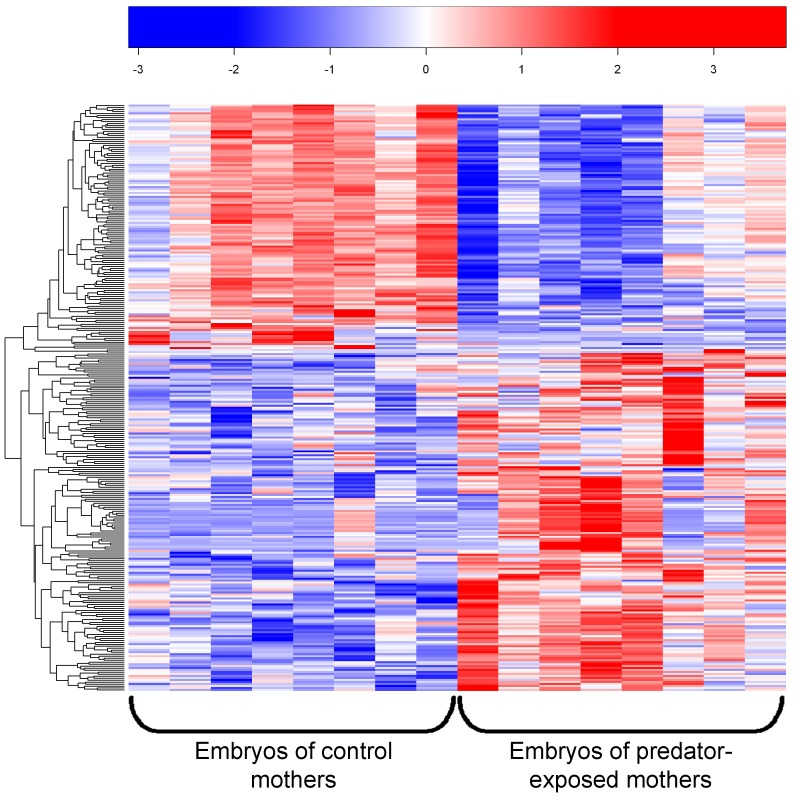
Exposing mothers to a predator led to an overall activation of genes in stickleback embryos. The heatmap shows the general pattern of gene regulation for the 295 genes differentially expressed (EdgeR) as a result of maternal exposure to predation risk. Red  =  upregulated, blue  =  downregulated compared to the mean value of a gene from all samples. Rows represent genes and columns represent clutches (one clutch per mother, n = 16 mothers total). log(CPM) data are normalized for library size and scaled so that every gene has the same mean (0) and standard deviation; units of change are standard deviations from the mean, and each row has the same range of color values.

There were n = 455 differentially expressed genes between embryos from control and predator-exposed mothers identified either by EdgeR, Cuffdiff, or both (Fig S1). Nearly twice as many genes were upregulated (n = 302) than downregulated (n = 153). A heatmap comparing the expression patterns within and between samples shows the same pattern; roughly 2/3 of differentially-expressed genes were upregulated and 1/3 downregulated in embryos of predator-exposed mothers compared to embryos of control mothers (Fig 1). All of the genes that were identified as differentially expressed in both EdgeR and Cuffdiff (n = 108) were expressed in the same direction between control and maternally-stressed offspring (i.e. the technique used to compare read counts between treatments did not influence their nature of gene expression difference), and there was a high correlation (R2 = 0.855) between the magnitude of fold change (FC) according to each method (Fig S2). In all there were 81 genes with greater than two-FC expression difference between treatments (22 downregulated and 59 upregulated in maternally-stressed offspring compared to control) and 32 genes with greater than five-FC difference (5 downregulated, 27 upregulated). A complete list of differentially-expressed genes and their expression levels is provided in Table S1. Principle component analysis separated maternal treatments along two principle components (Fig S3), accounting for 18.7% and 12.0% of the variation, respectively.

Using the human genome as background, GO enrichment analysis in DAVID revealed nine biological processes, seven cellular components, four molecular functions, and five KEGG pathways that were enriched in the dataset (FDR<0.05) (Table 1), including GO terms related to mitochondrial functioning, oxidative metabolism, neurological disease, and immune function. Changing the annotation and background from human to mouse and zebrafish tested whether the quality of between-species annotations within DAVID influenced the quality of GO and KEGG enrichment results. Using mouse annotations instead of human led to the inclusion of GO_BP terms associated with osmoregulation and coagulation, but did not largely change the GO_CC, GO_MF, or KEGG pathway annotations (Table S2). Using zebrafish annotations instead of human led to the inclusion of a new GO_BP term “generation of precursor metabolites and energy”, though this is not largely different from previously obtained GO_BP terms, and fewer terms met the FDR threshold (Table S3). The consistent recovery of GO and KEGG enrichment results across species, coupled with the failure of randomly-drawn gene lists to identify any significant GO terms, suggests that the results of the original DAVID analysis (Table 1) are robust and are not heavily biased by the use of human annotations and genome background.

**Table 1:** The most overrepresented biological annotations from the list of genes differentially expressed as a result of maternal exposure to predation risk.

Category	Term	Number of genes	Fold Enrichment	P-Value	FDR
GOTERM_BP_FAT	respiratory electron transport chain	12	9.7	3.40E-08	0.000057
	ATP synthesis coupled electron transport	11	10.1	9.80E-08	0.000170
	mitochondrial ATP synthesis coupled electron transport	11	10.1	9.80E-08	0.000170
	oxidation reduction	34	2.7	2.40E-07	0.000400
	cellular respiration	13	6.9	3.40E-07	0.000590
	electron transport chain	13	5.9	2.00E-06	0.003400
	oxidative phosphorylation	12	6.3	2.80E-06	0.004800
	cellular amino acid derivative metabolic process	15	4.7	4.00E-06	0.006900
	energy derivation by oxidation of organic compounds	13	4.7	2.20E-05	0.038000
					
GOTERM_CC_FAT	respiratory chain	13	9.3	1.20E-08	0.000016
	mitochondrial inner membrane	22	3.9	2.50E-07	0.000340
	mitochondrial membrane	25	3.4	3.10E-07	0.000410
	organelle inner membrane	22	3.6	8.40E-07	0.001100
	mitochondrial envelope	25	3.2	9.40E-07	0.001300
	mitochondrial respiratory chain	10	8.4	2.50E-06	0.003400
	mitochondrial membrane part	12	5.2	2.00E-05	0.027000
					
GOTERM_MF_FAT	heme-copper terminal oxidase activity	7	12.7	1.40E-05	0.021000
	oxidoreductase activity, acting on heme group of donors, oxygen as acceptor	7	12.7	1.40E-05	0.021000
	oxidoreductase activity, acting on heme group of donors	7	12.7	1.40E-05	0.021000
	cytochrome-c oxidase activity	7	12.7	1.40E-05	0.021000
					
KEGG_PATHWAY	Parkinson's disease	17	4.9	2.10E-07	0.000240
	Oxidative phosphorylation	17	4.9	2.60E-07	0.000300
	Complement and coagulation cascades	12	6.5	1.60E-06	0.001900
	Alzheimer's disease	17	3.9	5.50E-06	0.006400
	Huntington's disease	17	3.5	2.00E-05	0.023000

Genes were mapped to their associated biological process (BP_FAT), cellular component (CC_FAT), molecular function (MF_FAT), and KEGG pathways using *Homo sapiens* gene annotations and genome background within the DAVID Functional Annotation Tool (v6.7). Only terms with FDR <0.05 and ≥2 genes were included.

Pathway analysis in IPA revealed 13 clusters that represent cell proliferation, hematopoeisis, sensory organ development (especially eye development), adhesion of immune cells, kidney development, and metabolism of amino acids (Table 2).

**Table 2:** The most overrepresented biological functions (n≥10 molecules) from an enrichment analysis (Ingenuity Pathway Analysis software) of genes differentially expressed as a result of maternal exposure to predation risk.

Functional Annotation	# molecules	p-value	Sample molecules
quantity of cells	52	0.0032	ADD3, AGR2, AOC3, ATF4, BARHL2, C3, C6, CCND2, CD200, CISH, CP, CXCL12, CYP2J2, DNMT3B, EEF1D
quantity of blood cells	38	0.000463	ADD3, AGR2, ATF4, C3, C6, CCND2, CD200, CISH, CXCL12, EEF1D, ETV6, F2, FUT4, GNB3, HBA1/HBA2
morphology of embryonic tissue	22	0.00516	AHCTF1, DAD1, DRAP1, ETV6, F2, FGFR1, HES5, HIRA, HIST1H1C, HIST1H1D, HIST1H1E, KRT8, NCKAP1
development of sensory organ	20	0.00537	ATF4, BFSP2, CP, DNMT3A, FGFR1, GJA8, GNB3, GPX4, HES5, KNG1, LIM2, NEUROD4, POU3F4, PSAP, RARG
morphology of connective tissue	19	0.00419	ADD3, DNAJA1, ETV6, F2, HBA1/HBA2, HBB, HBD, HBZ, HIST1H1C, HIST1H1D, HIST1H1E, POU3F4, PSAP
eye development	18	0.00232	ATF4, BFSP2, CP, DNMT3A, GJA8, GNB3, GPX4, HES5, KNG1, LIM2, NEUROD4, PSAP, RARG, SOD1, SOD2
adhesion of tumor cell lines	15	8.89E-05	ALOX15B, APOH, B4GALNT2, CXCL12, CYP2J2, DBF4, F2, KNG1, NFKBIA, PXN, RAC1, RAP1GAP, S100P
aggregation of cells	14	0.00219	CP, CXCL12, ETV6, F2, KNG1, LGALS1, NFASC, NPHS1, PLCB2, PXN, SERPINC1, PERPIND1, VASP, VTN
adhesion of immune cells	14	0.0066	AOC3, APOH, C3, CD200, CXCL12, EZR, F2, FUT4, KNG1, NFKBIA, RAC1, RAP1GAP, VASP, VTN
morphology of the eye	13	0.00305	ATF4, BFSP2, CP, GJA8, GNB3, GPX4, HES5, NEUROD4, PSAP, RARG, SOD1, SOD2, VSX1
abnormal morphology of extraembryonic tissue	12	0.000529	DAD1, ETV6, F2, FGFR1, HIRA, HIST1H1C, HIST1H1D, HIST1H1E, POU2F1, PXN, TFPI, YAP1
kidney development	11	0.0105	EZR, FABP2, FGFR1, HBB, IGFBP2, LCAT, ODC1, PSAP, SOX4, TFAP2B, TKT
metabolism of amino acids	10	0.0006	ARG2, ATF4, BHMT, BHMT2, CKB, CXCL12, GPT, HAL, QDPR, TDO2

# molecules  =  the number of unique RNA molecules (i.e. differentially expressed genes) associated with a function within the IPA database; p-value  =  the enrichment p-value for the function; Sample molecules  =  a sample of genes associated with the function that were differentially expressed in maternally-stressed embryos compared to embryos of control mothers in the direction of regulation expected for association with the biological function.

### Metabolism genes

Several genes involved in oxygen transport, ATP synthesis, and other metabolic processes were significantly upregulated in maternally-stressed embryos including embryonic hemoglobin alpha (hbae1) and beta (hbbe1.1), all of the major cytochrome c oxidase subunits (COX1,2,3,4i2,5b,6a2,7c), cytochrome b (CYTB), and lactate dehydrogenase D (LDHD). The gene encoding thyroid hormone receptor interactor 13 (TRIP13) was downregulated.

### Neural development genes

**Figure 2:**
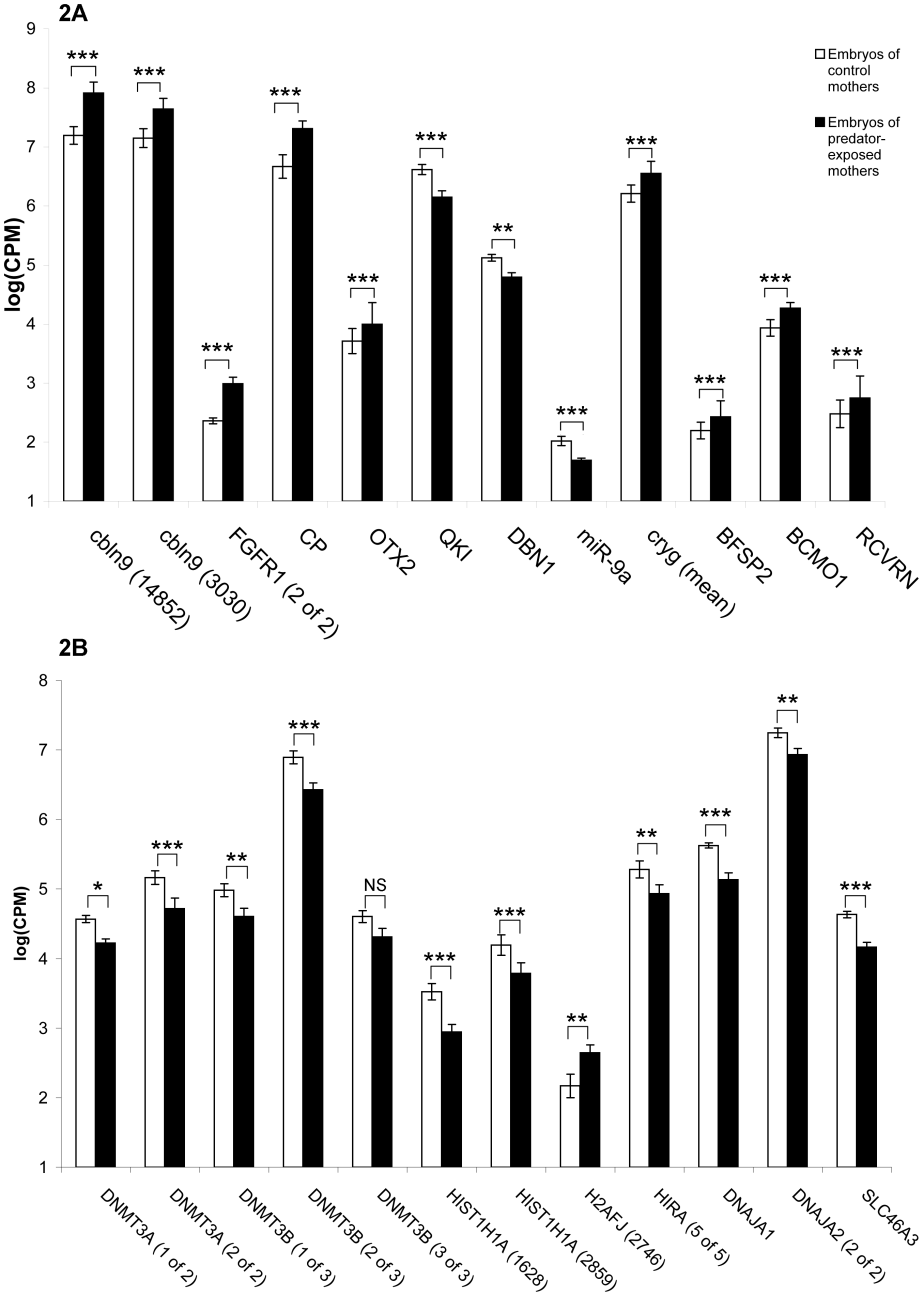
Maternal exposure to predation risk altered expression of embryonic genes involved in neural growth and epigenetic modification. A) The expression of DNA methyltransferase genes, histone genes, and genes that interact with histone and DNA methylation in 3dpf stickleback embryos from mothers exposed to a predator and control mothers. B) The expression of genes involved in the proliferation and differentiation of neurons. Each bar represents the mean ± standard error of the logarithm of adjusted Counts Per Million mapped reads across clutches of embryos from n = 8 mothers, and 10 pooled embryos per clutch. NS = raw p-value>0.05, *p<0.05, **p<0.01, ***p<0.001.

Numerous genes involved in the differentiation of neurons and the formation and survival of neurites were differentially expressed between maternal treatments (Fig 2a). These included upregulation of two genes that encode cerebellin 9 (cbln9), a gene encoding the fibroblast growth factor receptor 1 (FGFR1), ceruloplasmin (CP), and orthodenticle homeobox 2 (OTX2), and downregulation of quaking (QKI), drebrin (DBN1), and the microRNA miR-9a (ola-mir-9a-1). Genes involved in eye formation were also upregulated, including ten genes coding for gamma crystallin (crygm).

### Glucocorticoid-associated genes

In three day post-fertilization sticklebacks the HPI axis is not yet active (Paitz et al, unpublished). Therefore we did not expect to observe the differential expression of genes related to cortisol synthesis in this study. As predicted, several genes central to HPI axis function were not expressed at all (i.e. all samples <1 CPM), including corticotropin-releasing hormone (CRH), the CRH receptor 1 (CRHR1), one gene encoding proopiomelanocortin (POMC), the adrenocorticotropic hormone receptor (MC2R), and one of the genes encoding 3-β-hydroxysteroid dehydrogenase (HSD3B2), which is involved in the de novo synthesis of cortisol. Interestingly, however, some genes related to HPI axis functioning were expressed at low levels but were not significantly different between maternal treatments, including the second gene encoding POMC, and the genes encoding proprotein convertase 1 (PCSK1) and steroidogenic acute regulatory protein (star). Several genes involved in the production of cortisol precursors, and the reception and metabolism of cortisol were expressed at detectable levels but were not differentially expressed between maternal treatments, including one of the genes encoding 3-β-hydroxysteroid dehydrogenase (HSD3B7), the genes encoding 11β-hydroxysteroid dehydrogenase 2 (HSD11B2) and 3 (11-beta-hsd3), and the two genes that code for glucocorticoid receptor (NR3C1). There was one gene, the FK506 binding protein 4 (FKBP4), that was differentially expressed (downregulated in maternally-stressed embryos). Also differentially expressed in maternally-stressed embryos were genes that are known to have altered expression in tissues treated with the synthetic glucocorticoid dexamethasone, including downregulation of C3 and retinoic acid receptor gamma (RARG), and upregulation of SOD1, CISH, NFKBIA, and IGFBP2.

Glucocorticoids can affect immunity [43], [59] and as expected, many genes involved in the immune response were differentially expressed in maternally-stressed embryos. Four genes encoding complement component 3 (C3) were significantly downregulated in maternally-stressed embryos, but a different two C3 genes were upregulated. Among others upregulated were complement component 6 (C6), 8B (C8B), and NFKB-inhibitor alpha (NFKBIA). Genes that were downregulated included immunoglobulin heavy constant mu (IGHM) and NLR family member X1 (NLRX1).

### Accelerated development genes

We detected the differential expression of several genes involved in normal development, including genes responsible for the genome-wide epigenetic control of gene expression. Several genes involved in the maintenance of epigenetic modifications (Fig 2b) were differentially expressed, including two DNA methyltransferases (DNMT3A and DNMT3B), and three genes encoding histone proteins (HISTH1A, H2AFJ).

Interestingly, genes involved in sexual differentiation were also differentially expressed between treatments. Enzymes responsible for the metabolism of sex steroids were upregulated including steroid sulfatase (STS) and 17β hydroxysteroid dehydrogenase 10 (HSD17B10). Gonadotropin-releasing hormone receptor 1 (GNRHR1), responsible for the reception of GNRH and subsequent release of LH and FSH during sexual maturation, was downregulated. Also upregulated were the male sex-determining protein (DMRT1Y) and one gene encoding sex determining region Y-box 4 (SOX4).

### Non-coding RNAs

Several non-coding RNAs were differentially expressed in embryos as a result of maternal exposure to predation risk including upregulation of many small nucleolar RNAs (SNORA 3, 18, 23, 73 and 74; SNORD 15, SNOU 1, 3, 4 and 85), upregulation of two novel microRNAs (ENSGACG00000022837, ENSGACG00000022725), and downregulation of the microRNA miR-9a.

### Embryo morphology

**Figure 3:**
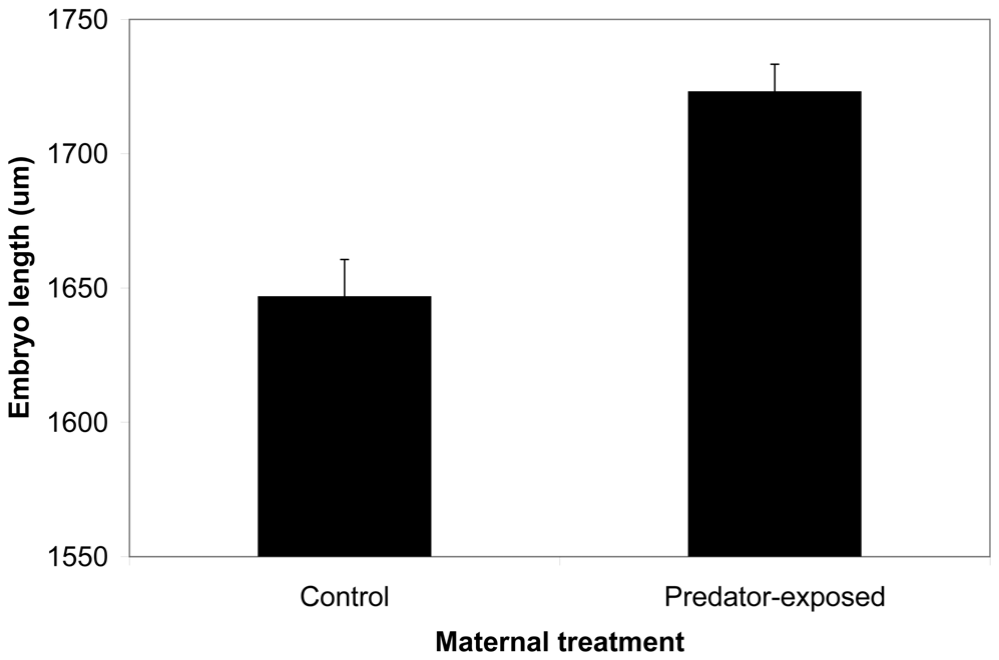
Embryos of stickleback mothers exposed to predation risk were longer than embryos of control mothers. Three days post-fertilization embryos of control mothers (n = 127 embryos) and mothers that were chased with a model predator (n = 388 embryos) were measured along the anterior-posterior axis. Embryos of predator-chased mothers were significantly longer than control mothers (F_1,14.4_ = 5.241, p = 0.038).

Embryos of mothers exposed to predation risk were longer along the anterior-posterior axis (1723.0±10.3 um, mean ± standard error) than embryos of control mothers (1646.7±13.9 um; F_1,14.4_ = 5.241, p = 0.038; Figure 3). We did not detect an effect of maternal predator exposure on embryonic eye diameter (offspring of predator-exposed mothers: 393.0±3.4 um, offspring of control mothers: 385.5±5.3 um; F_1,16.1_ = 0.629, p = 0.439).

## Discussion

This unbiased genome-wide expression survey identified some of the molecular mechanisms in embryos that respond to maternal experience. In general, maternal exposure to predation risk had an activating effect on offspring genome-wide expression and affected biological pathways involved in energy homeostasis, proliferation of cells, production of neurites and blood cells, differentiation of sensory neurons, and immunity. Many of these results are consistent with previous findings in sticklebacks and other animals that associated maternal stress with increased offspring growth [22], [43] and metabolism [33], altered immune function [59] and behavior [20], [33], [36]. Our data suggest that when mothers are exposed to a stressor, a shift occurs at the molecular level in offspring resulting in a divergence in the developmental program. Further, embryos of predator-exposed mothers were larger, either because they began with more resources (as suggested by [33]) or grew more quickly during early development. Many of the physiological systems involved in the embryonic response to maternal exposure to predation risk are evolutionarily conserved between sticklebacks and mammals [17]–[18], amphibians [3], [23], reptiles [19] and birds [7], [60]. Therefore our results identify molecular mechanisms in offspring that might be altered by maternal experience in other organisms.

### Maternal predator exposure accelerates metabolism, growth and neural development

We detected a significant effect of maternal exposure to predation risk on several genes involved in metabolic processes, including upregulation of genes involved in the delivery of oxygen to tissues, the production of ATP, anaerobic metabolism, and the metabolism of amino acids and lipids. Coupled with the upregulation of many genes involved in the increased proliferation of cells and a previous finding that maternally-stressed stickleback embryos are larger and consume more oxygen [33], these findings suggest that maternal experience with predators accelerates offspring growth.

Maternal exposure to predation risk upregulated several genes involved in the growth, survival, and death of neurites, the differentiating axons and dendrites that newly-forming neurons use to contact one another (reviewed in [61]) in offspring. These include genes involved in synapse formation in cortical neurons [62], the balance between neuronal survival and death during inflammation [63], and the development of sensory organs, fore-brain, mid-brain and pituitary gland [64]. Together these results suggest that exposing a mother to predation risk accelerates the proliferation and differentiation of neurons in her developing embryos. Early changes in the development of the brain and eyes could be a mechanism by which maternal exposure to predation risk influences offspring learning [35] and behavior [33], [36] in sticklebacks.

Genes involved in the formation and use of the eye were upregulated, including genes involved in formation of the lens [65] and neurites [66], differentiation of fiber cells in the lens [67], and phosphorylation of rhodopsin in the retina [68]. Sanogo et al. [42] measured gene expression changes in the brains of adult sticklebacks exposed to predator cues and found differential regulation of several genes involved in photoreception and phototransduction. Our results suggest that, at the molecular level, the developing embryo visual system might respond in a similar way to indirect exposure to predation risk (via mothers) that the adult visual system does to direct exposure to predation risk. We did not detect a difference in eye diameter between embryos of maternally-stressed and control mothers, possibly due to limitations in our method of measurement, limited statistical power to detect a subtle difference, or a true lack of difference between the embryos. Future studies wishing to understand the influence of maternal stress on the developing embryo visual system might focus on the proliferation and differentiation of cells of the visual system and the localization of the expression of the specific genes reported here.

### Effects of maternal predator exposure on stress- and immune-related genes

Many of the genes that are essential for the functioning of the vertebrate HPI axis were not expressed in 3dpf stickleback embryos, including several key neuroendocrine ligands, their receptors, and enzymes responsible for the production of cortisol. These results are not surprising given results that suggest rapid clearance of embryonic steroids early in development (Paitz et al, unpublished). Also, we detected a significant effect of maternal stress on the expression of genes shown to be similarly influenced in humans and other mammal cells by exposure to the synthetic glucocorticoid dexamethasone. It is therefore possible that some of the genes differentially expressed in stickleback embryos in response to maternal stress were activated or repressed through the action of maternal cortisol, though cortisol was not measured in this experiment. Further study is needed to understand the magnitude and tissue specificity by which maternal glucocorticoids might influence the embryonic transcriptome.

Consistent with the known effects of glucocorticoids on immune function, we detected the differential regulation of several immune genes, including genes involved in the innate and adaptive immune responses. Previous studies in humans [69] and other primates [70] have found an enhancing effect of maternal stress on the offspring innate immune response, coupled with a depression of the adaptive immune response [71].

### Effects of maternal predator exposure on genes associated with sexual differentiation

Sexual differentiation in sticklebacks does not begin until the primordial germ cells (PGCs) within the gonads of females begin to proliferate at 11 days post-fertilization [51]. An unexpected result of this study, then, is that pathways involved with gonadal steroids and sexual differentiation were influenced by maternal exposure to predation risk in three days post-fertilization embryos. Sticklebacks are external fertilizers and stickleback males are the heterogametic sex, so it is interesting that maternal stress would affect sexual differentiation when sex is not determined until after mothers have broken contact with offspring. These results suggest that sexual maturation and gonadal differentiation could be occurring earlier in maternally-stressed embryos, again reflecting a difference in the timing of development.

### Maternal predator exposure influences genes associated with epigenetic modifications

Several factors involved in the epigenetic silencing of gene transcription, including multiple DNA-methyltransferases and two histone proteins, were differentially expressed in maternally-stressed embryos. DNMT3 is responsible for de novo methylation, specifically methylation at CpG sites, during development [72]. Decreased methylation of CpG sites in or nearby genes is associated with increased transcription [49] and so downregulation of DNMT3A and DNMT3B, as we observed in this study, is expected to increase activation of genes with nearby CpG islands. Further, we observed a downregulation of the histone H1 and H2A proteins, which are part of the histone complex that interacts with methylated DNA to suppress transcription (reviewed in [73]). Downregulation of these genome-wide epigenetic repressors of transcription is consistent with the finding that twice the number of genes were activated (i.e. upregulated) in response to maternal stress than were repressed (i.e. downregulated). These results are consistent with the emerging literature on epigenetic modifications to the genome that have long term effects on offspring development (e.g. [41]).

### Maternal predator exposure influenced the expression of microRNAs

The unbiased whole transcriptome approach we used allowed us to identify the differential expression of several small RNAs that are not picked up in traditional microarray studies. We identified several microRNAs differentially expressed between maternally stressed and control embryos including several small nucleolar RNAs (SNORA3,18,23,73,74, SNORD15, and SNOU85). Some snoRNAs are thought to regulate specific alternatively spliced transcripts [74], suggesting they could modulate the relative abundance of alternative transcripts of genes.

We also detected several novel differentially expressed miRNAs including one (ENSGACG00000022837) that was expressed more than 193-fold higher in maternally-stressed embryos than control embryos (FDR  = 0.003). It is possible that some of these known or novel miRNAs are involved in the epigenetic transfer of maternal stress to offspring. For example a miRNA in zebrafish (miR-430) accelerates the deadenylation of maternal mRNAs during embryonic development, promoting their clearance from embryos [75]. Also, in mice, a miRNA (miR-124) is involved in the epigenetic control of embryonic growth rate [76].

## Conclusion

These results suggest that early stickleback embryos respond to maternal exposure to predation risk via changes in gene expression. Embryos of predator-exposed mothers had greater expression of genes involved in neurite formation, the development of the visual system and aerobic and anaerobic metabolism. Embryos of predator-exposed mothers were also larger. Together these results suggest that offspring of mothers exposed to predation risk undergo a general acceleration of the developmental program. Given that a faster life history is associated with high predation environments [45]–[47], offspring of predator-exposed mothers might be more likely to survive to reproduce in a high predation environment. Further study is needed to elucidate the myriad molecular interactions between genes that are differentially-regulated as a result of maternal exposure to predation risk and to understand their relationships to previously-observed maternal effects in this system [33]–[36].

## Supporting Information

Figure S1 The intersection of upregulated, downregulated, and total differentially expressed genes as identified in EdgeR and Cuffdiff from the same alignment of reads to the stickleback reference genome (n = 455 total unique genes, 302 upregulated, 153 downregulated).
(DOC)

Figure S2 comparison of fold change data of n = 108 genes identified via both Bowtie-edgeR and Cufflinks-Cuffdiff analyses as differentially expressed in stickleback embryos exposed to maternal stress. While Cuffdiff generally called a higher fold change than edgeR (slope  = 0.88), there was nevertheless a tight correlation between the two (R-square  = 0.855). All genes were found to be upregulated (quadrant I) or downregulated (quadrant III) in both analyses.
(DOC)

Figure S3 A Principle Components Analysis of general gene expression patterns of each sample as determined by RNA-seq and edgeR differential expression analysis. Blue squares represent embryos of mothers exposed to predation risk and red circles represent embryos of control mothers. Each data point represents total RNA from 10 pooled embryos from a single mother.
(DOC)

Figure S4 A diagram illustrating the measurements taken of stickleback embryo length (A) and eye diameter (B). Morphological data were then compared between embryos of mothers exposed to a predator and embryos of control mothers.
(DOC)

Table S1 The stickleback genes found to be differentially expressed in 3dpf embryos of mothers exposed to a predator compared to embryos of control mothers. Direction: “up”  =  higher expression in embryos of mothers exposed to a predator compared to embryos of control mothers, “down”  =  lower expression in embryos of mothers exposed to a predator compared to embryos of control mothers. FDR  =  false discovery rate (p-value corrected for multiple testing), logCPM  =  log(adjusted counts per million reads) from edgeR analysis, FPKM  =  fragments per kilobase of exon per million mapped reads, the count method used in Cufflinks analysis, “shared?”  =  whether a gene was identified as differentially expressed using both EdgeR and Cuffdiff analyses (yes) or in one analysis but not the other (blank).
(XLS)

Table S2 The most overrepresented biological annotations from the list of genes differentially expressed between 3dpf stickleback embryos exposed to maternal stress and embryos of control mothers as measured via RNA-seq. Genes were mapped to their associated biological process (BP_FAT), cellular component (CC_FAT), molecular function (MF_FAT), and KEGG pathways using *Mus musculus* gene annotations and genome background within the DAVID Functional Annotation Tool (v6.7). Only terms with FDR <0.05 and ≥2 genes were included.
(XLS)

Table S3 GO terms and KEGG pathways obtained using *Danio rerio* gene annotations and genome background within the DAVID Functional Annotation Tool (v6.7). Only terms with FDR <0.05 and ≥2 genes were included.
(XLSX)
