# Supplementary material for: Maternal Experience with Predation Risk Influences Genome-Wide Embryonic Gene Expression in Threespined Sticklebacks (Gasterosteus aculeatus)
Source: PLoS One. 2014 Jun 2;9(6):e98564. doi: 10.1371/journal.pone.0098564 (PMC4041765; doi:10.1371/journal.pone.0098564)
Supplement: Figure S1 — The intersection of upregulated, downregulated, and total differentially expressed genes as identified in EdgeR and Cuffdiff from the same alignment of reads to the stickleback reference genome (n = 455 total unique genes, 302 upregulated, 153 downregulated). (DOC) [file pone.0098564.s001.doc]

**130**

**82**

**90**

**UP**

**30**

**26**

**97**

**DOWN**

Cuffdiff

EdgeR

Cuffdiff

EdgeR

**TOTAL**

**EdgeR**

**Cuffdiff**

**160**

**108**

**187**
