# Supplementary material for: Maternal Experience with Predation Risk Influences Genome-Wide Embryonic Gene Expression in Threespined Sticklebacks (Gasterosteus aculeatus)
Source: PLoS One. 2014 Jun 2;9(6):e98564. doi: 10.1371/journal.pone.0098564 (PMC4041765; doi:10.1371/journal.pone.0098564)
Supplement: Figure S2 — comparison of fold change data of n = 108 genes identified via both Bowtie-edgeR and Cufflinks-Cuffdiff analyses as differentially expressed in stickleback embryos exposed to maternal stress. While Cuffdiff generally called a higher fold change than edgeR (slope = 0.88), there was nevertheless a tight correlation between the two (R-square = 0.855). All genes were found to be upregulated (quadrant I) or downregulated (quadrant III) in both analyses. (DOC) [file pone.0098564.s002.doc]

**I**

**II**

**III**

**IV**
