# Supplementary figures and images for: Maternal Experience with Predation Risk Influences Genome-Wide Embryonic Gene Expression in Threespined Sticklebacks (Gasterosteus aculeatus)
Source: PLoS One. 2014 Jun 2;9(6):e98564. doi: 10.1371/journal.pone.0098564 (PMC4041765; doi:10.1371/journal.pone.0098564)

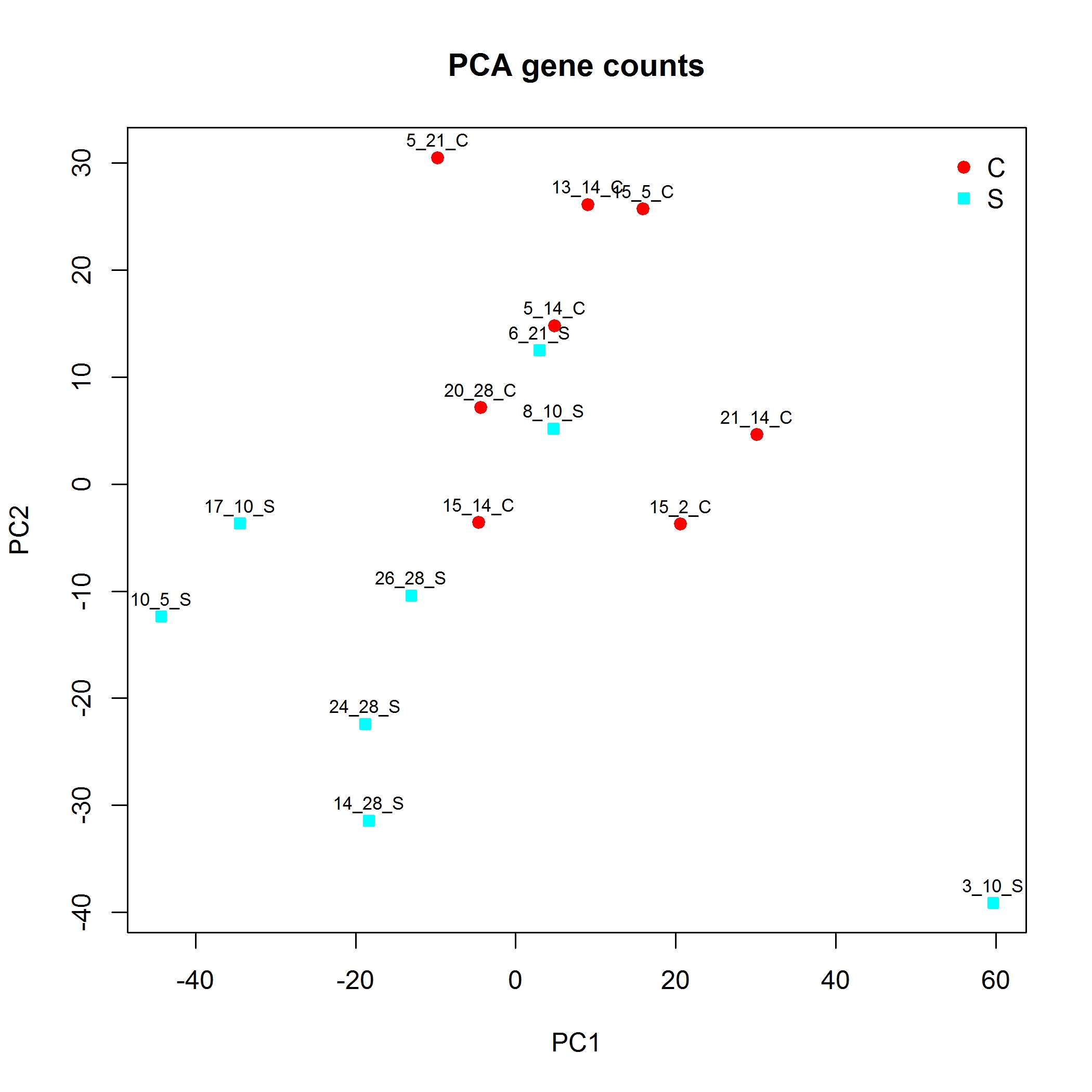

Supplement: Figure S3 — A Principle Components Analysis of general gene expression patterns of each sample as determined by RNA-seq and edgeR differential expression analysis. Blue squares represent embryos of mothers exposed to predation risk and red circles represent embryos of control mothers. Each data point represents total RNA from 10 pooled embryos from a single mother. (DOC) [file pone.0098564.s003.doc]

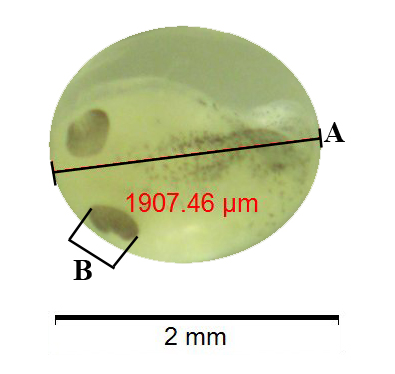

Supplement: Figure S4 — A diagram illustrating the measurements taken of stickleback embryo length (A) and eye diameter (B). Morphological data were then compared between embryos of mothers exposed to a predator and embryos of control mothers. (DOC) [file pone.0098564.s004.doc]
